# Estimating the severity of COVID-19: Evidence from the Italian epicenter

**DOI:** 10.1371/journal.pone.0239569

**Published:** 2020-10-01

**Authors:** Paolo Buonanno, Sergio Galletta, Marcello Puca

**Affiliations:** 1 Department of Management, Economics & Quantitative Methods, University of Bergamo, Bergamo, Italy; 2 Center for Law and Economics, ETH Zürich, Zürich, Switzerland; 3 Walker School of Business and Technology, Webster University Geneva, Bellevue, Switzerland; Boston University, UNITED STATES

## Abstract

We provide results on the level of COVID-19 excess mortality in the Italian region of Lombardy and in the province of Bergamo using official and original data sources. Since February 2020 Lombardy and in particular the province of Bergamo have been severely hit by the novel COVID-19 infectious disease. Combining official statistics, retrospective data and original data (i.e., obituaries and death notices) we provide a tentative estimate of the number of deaths either directly or indirectly, associated with COVID-19 as well as the total number of persons infected. Our findings suggest that the reported number of deaths attributable to COVID-19 identified by public authorities accounts only for one half of the observed excess mortality between March 2020 and previous years.

## 1 Introduction

The Lombardy region has been at the epicenter of the novel coronavirus (COVID-19) Italian epidemic. Since the detection in Rome of two COVID-19 positive tourists traveling from Wuhan on January 31, 2020, and the early detection of several cases in the northern area of the country, the disease spread for almost two months without much public intervention. On February 23, 2020, the first COVID-19–associated death was recorded in an hospital of Bergamo, a Lombardy’s province. Despite the worrying signals, national politicians, public authorities, and local business industry confederations were dismissing the risks of COVID-19. See [1] for a detailed timeline of the key events of the COVID-19 Italian outbreak. By the end of March 2020, official reporting from national public authorities, available from the Civil Protection Department data website https://github.com/pcm-dpc/COVID-19/tree/master/dati-regioni, counted 7,593 COVID-19–associated deaths and 44,773 infected individuals, respectively the 57.7% and 40.5% of all the Italian cases.

Initial contributions have assessed the severity of COVID-19 providing either “crude” or adjusted-biased case fatality rate (CFR) measure based on model on infection dynamics [2–5]. Broadly speaking, these measures account for the share of deaths linked to a specific disease in comparison with the total number of individuals that have been diagnosed with that disease in a given period, and can be interpreted as the probability that a patient dies from the disease. These measures are important from an epidemiological point of view, since they allow an assessment of the healthcare capacity in response to an outbreak. However, these measures have limitations. In particular, there would be clear biases if one uses the aggregate numbers of reported cases and COVID-19–associated deaths to compute CFR estimate to understand the severity of the disease [6, 7].

On the one hand, there might be an underestimation of the intensity during the first phases of an epidemic if several days pass between the moment an individual experiences the first symptoms and the day health authorities detect the infection, causing a *censoring bias* [8–10]. On the other hand, there might be an overestimate of the fatality rate if health authorities tend to concentrate only on severe cases, resulting in poor information on milder cases and causing an *ascertainment bias*. Similar bias will be present if the health-care system has reached its capacity constraint and is not able to detect further cases regardless of the severity. Adjusted estimates might as well face measurement issues as they mainly rely on the underlying assumptions on how to attenuate the bias. Despite the possible adjustments, however, these limitations make the CFR only a partial guidance to policymakers, mostly because it does not clearly reveal the actual consequences of an outbreak on the general population. To provide a more reliable evaluation of the severity of a disease in the general population it is common in the epidemiological literature to focus on the excess of mortality. This is usually measured looking at the rates of death in the population and compare it with a seasonal baseline level referring to periods in which the disease was not circulating [11–13].

Our contribution is to provide a first estimate of the excess of mortality rate in the general population in the Italian region of Lombardy during the months of the COVID-19 outbreak, and to assess the validity and the practical reliability of official figures combining different data sources. Specifically, we compare the official number of all-cause deaths reported in March 2020 with those reported in the same month of the previous year in a sample of Lombardy’s municipalities. Next, with this result in hand, we recover the expected number of total persons infected for different levels of hypothetical CFR. These results add to the recent evidence on the excess of mortality associated with COVID-19 using data from other countries [14, 15]. In addition, we show that the number of deaths associated to COVID-19 identified by public authorities are only 50% of the observed excess mortality rate between March 2020 and March 2019. Finally, we confirm this evidence by using an alternative method that we propose as a valid substitute for the use of unreliable (or unavailable) official data about population death rate.

## 2 Methods

### Data collection

We collected data from several sources. We create a measure of excess of mortality which compares the number of all deaths observed in March 2019 to those of March 2020 for a sample of Italian municipalities in the region of Lombardy. The Italian National Statistical Institute (ISTAT) released information on the mortality rates for 548 of the 1,507 total Lombardy’s municipalities, accounting for roughly 67% of Lombardy’s total population. Data have been released on April 9, 2020. The population coverage at the provincial level for Lombardy province is: 72.66% for Bergamo, 67.61% for Brescia, 51.58% for Como, 73.38% for Cremona, 55.76% for Lecco, 75.98% for Lodi, 67.17% for Mantova, 83.97% for Milan, 60.93% for Monza-Brianza, 51.77% for Pavia, 47.23% for Sondrio and 25.54% for Varese. The sample contains municipalities which, between January 1 2020 and March 28 2020, registered an increase of the mortality rate of at least 20% compared to the period 2015-2019. For more information see https://www.istat.it/it/archivio/240401. We merge this information with data on COVID-19 associated deaths at the municipality level obtained from the Lombardy Region website on March 30, 2020. Data refer to the status reported by regional public authorities on the March 30, 2020. The list contains, for each Lombardy’s municipality, the number of dead patients who tested positive during hospitalization or *post mortem*. We use these variables in section 3.1, to compare the reported number of COVID-19 associated deaths with the actual number of excess of deaths observed in the same period.

In section 3.1.1 we use municipal mortality data that were collected through a survey sent by the two main local newspapers *L’eco di Bergamo* and *Giornale di Brescia* and the data analysis firm inTwig to all register offices of municipalities belonging to Bergamo and Brescia provinces. As of March 31, 2020, our final dataset contains 332 municipalities, of which 139 (resp. 193) refer to Bergamo (resp. Brescia) province, and represent 71% (resp. 95%) of the total province population.

In Section 3.3 we use an alternative measure of mortality rates. We digitalized obituaries published by *L’Eco di Bergamo* that is the most read and circulated daily newspaper in the province of Bergamo. Our final dataset contains 3,083 unique individuals from March 1 to March 31, 2020.

Finally, in Section 3.4, we collected data for individuals who tested positive to COVID-19 in the Italian region of Lombardy in March 2020. The available data do not identify the actual day of death. We only observe whether an individual in the sample was dead or alive at the moment we collected the data, i.e., on March 31, 2020. The dataset contains a total of 43,176 cases.

### Hypothetical case fatality rate

To assess the actual infection rate, in section 3.2 we perform a back-of-the-envelope computation using different fatality ratios as reported in [5] (Table 1 of the cited manuscript) for each age group in Lombardy. For details about the modelling assumptions which are assumed for the infection dynamics and for modelling the relations between infection and mortality rates one can refer to [2] and [5] methodological sections. Under the restrictive assumption that infections follow a uniform distribution across each age group and that the age-specific theoretical fatality ratios reported in [5] are valid also for Lombardy, we compute the hypothetical fatality rate multiplying the theoretical fatality ratio (first column of Table 1) by the share of population for each age group (second column of Table 1). We report the results of this computation in the last column of Table 1. Data on age groups are provided by the Italian National Statistical Institute at https://www.istat.it/en/population-and-households?data-and-indicators. Next, summing up together all the elements of the third column of Table 1, we find an aggregate hypothetical fatality rate of approximately 1.5. Finally, in Section 3.2, we use this hypothetical mortality rate to compute the share of population likely to be infected, by dividing for each province the excess mortality rate (i.e., excess mortality over total population in 2019) by 0.015.

**Table 1:** Hypothetical fatality rate, by age group.

Age group	Fatality rate %	Lombardy population %	Hypothetical Fatality Rate
0-9	0	9.2	0
10-19	0.01	9.4	0.00094
20-29	0.03	9.6	0.0028
30-39	0.08	12.3	0.00984
40-49	0.15	16.4	0.0246
50-59	0.6	15	0.09
60-69	2.2	11.8	0.2596
70-79	5.1	9.7	0.4947
80+	9.3	6.6	0.6138
		100%	1.496

*Notes*: This table reports the hypothetical fatality rates computed as the product of theoretical fatality rates reported in [5] times the share of each age group over total population in Lombardy.

## 3 Results

### 3.1 How severe is under-reporting in COVID-19 fatality rate and diffusion in Lombardy?

**Fig 1:**
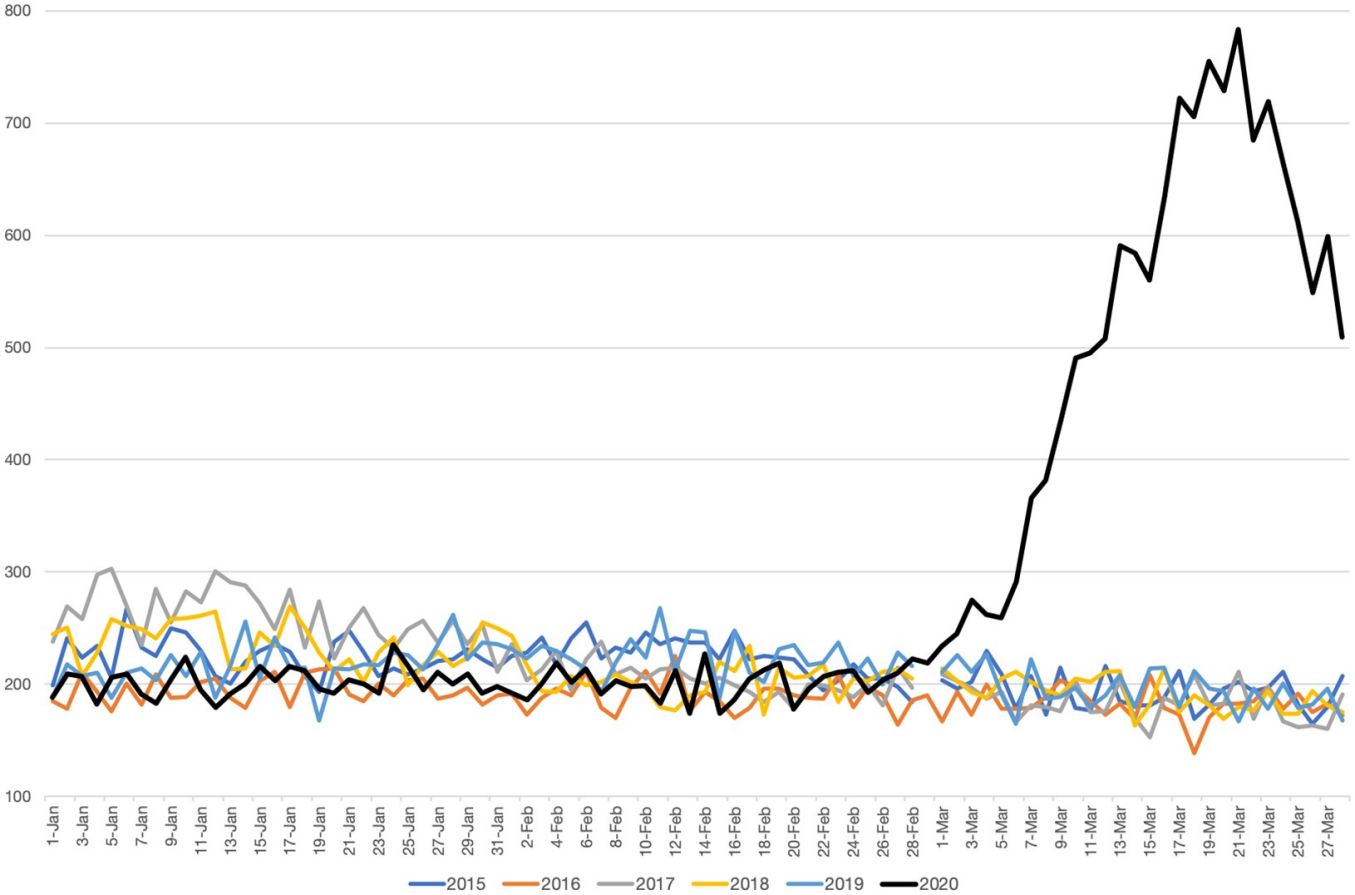
Daily numbers of deaths in Lombardy from January, 1 to March, 28 (2015-2020). *Notes*: Data refer to the daily numbers of deaths in the 548 Lombardy’s municipalities comprised in the ISTAT release from January 1 to March 28 for each year from 2015 to 2020.

**Comparing fatality rates across years**. Table 2 reports basic summary statistics for the municipalities in our sample. At the aggregate level, we report an increase going from 5,438 deaths in March 2019 to 14,613 in March 2020, consisting of a 285% growth of the 2020 average mortality rate compared to 2019. For each municipality, we compute the mortality rate as the number of reported deaths over the total 2019 population. We obtain similar figures when we compute mortality rates using the average population observed over the 2012-2018 period. As of March 28, 2020, however, only 4,712 deaths have been registered by the regional government as COVID-19 –associated deaths in those municipalities. This implies that number of fatalities attributed to COVID-19 only account for approximately one half of the observed excess mortality between March 2020 and March 2019. We observe similar under-reporting figures when we compare March 2020 fatalities with the average fatalities observed between 2012 and 2018. In Fig 1 we plot the evolution of daily deaths in Lombardy from January, 1 to March, 28 for each year from 2015 to 2020. As it clearly emerges, the number of deaths has experienced a consistent increase in March 2020 with respect to previous years.

**Table 2:** Summary statistics, by municipality.

	Obs.	Mean	Std. dev.	Min	Max
Variables	(1)	(2)	(3)	(4)	(5)
**Panel A**. *Data at the 31st of March*					
Population March 2019	332	6,022	13,372	88	198,300
Population March 2012-2018	332	5,982	13,107	86	194,503
COVID Deaths	332	8.880	18.79	0	220
Total Deaths March 2019	332	4.925	13.11	0	194
Total Deaths March 2020	332	23.13	47.19	0	602
Excess Mortality	332	18.21	35.75	-2	477
Excess Unexpected Mortality	332	9.328	18.96	-12	267
**Panel B**. *Data at the 28th of March*					
Population March 2019	548	12,271	60,520	508	1,380,197
Population March 2012-2018	548	12,066	58,399	247.8	1,330,148
COVID Deaths	548	8.599	20.67	0	330
Total Deaths March 2019	548	9.923	48.70	0	1,100
Total Deaths March 2020	548	26.67	76.69	2	1,551
Excess Mortality	548	16.74	35.22	-4	474
Excess Unexpected Mortality	548	8.144	17.38	-10	285

*Notes*: This table reports summary statistics of the variables used in our analysis. Panel A refers to all municipalities of Bergamo and Brescia provinces in the sample data available at the 31st of March. Panel B refers to all municipalities of Lombardy in the sample data available at the 28th of March.

**Fig 2:**
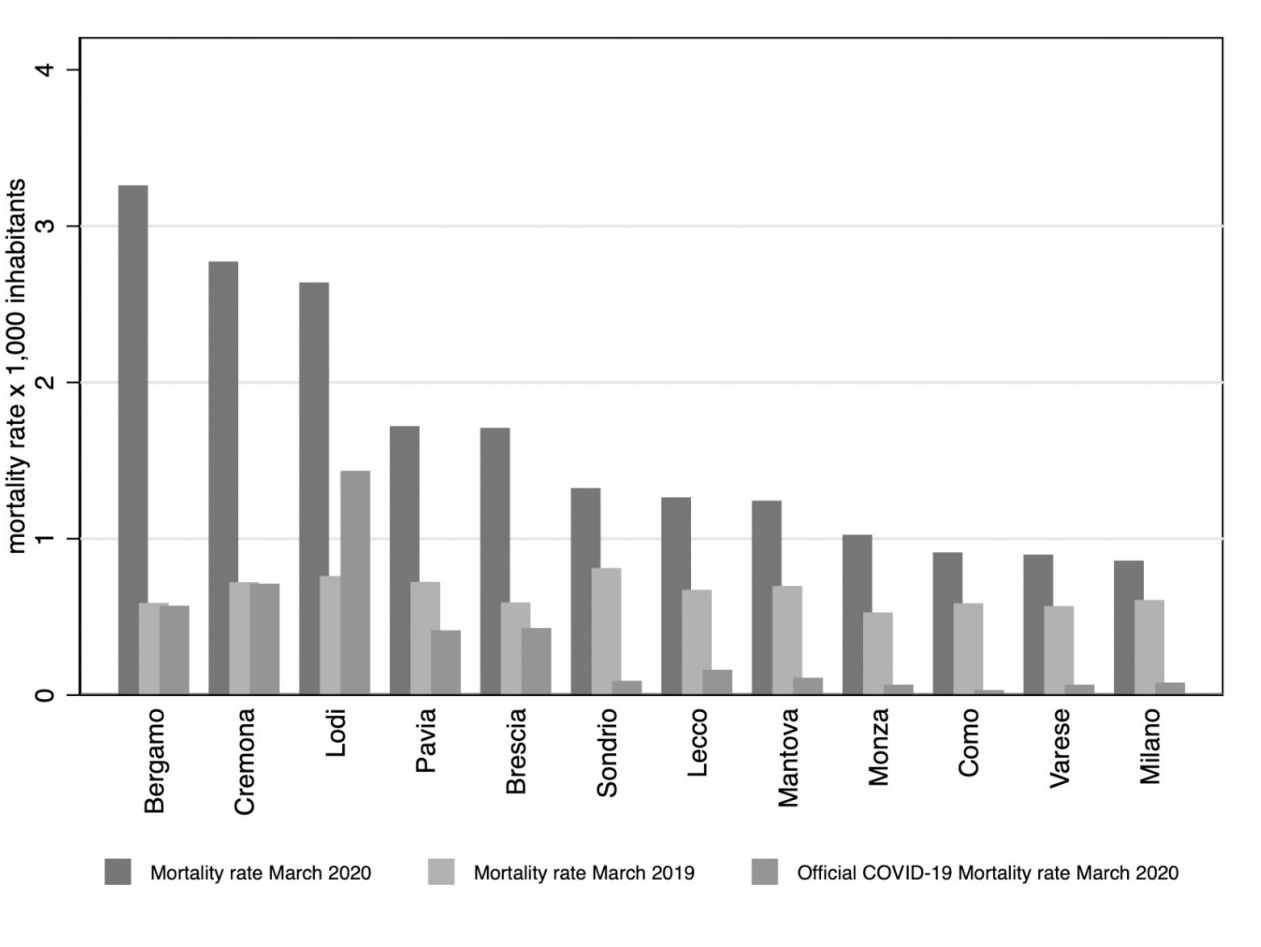
Distribution of mortality rates, by province. *Notes*: This graph reports the actual mortality rate in March 2020 and March 2019 as well as the reported mortality rate of COVID-19 as detected by the health authorities, by provinces. Data refer to the month of March until the 28th. These measure are based on our sample of 548 municipalities covering 67% of the regional population.

**Fig 3:**
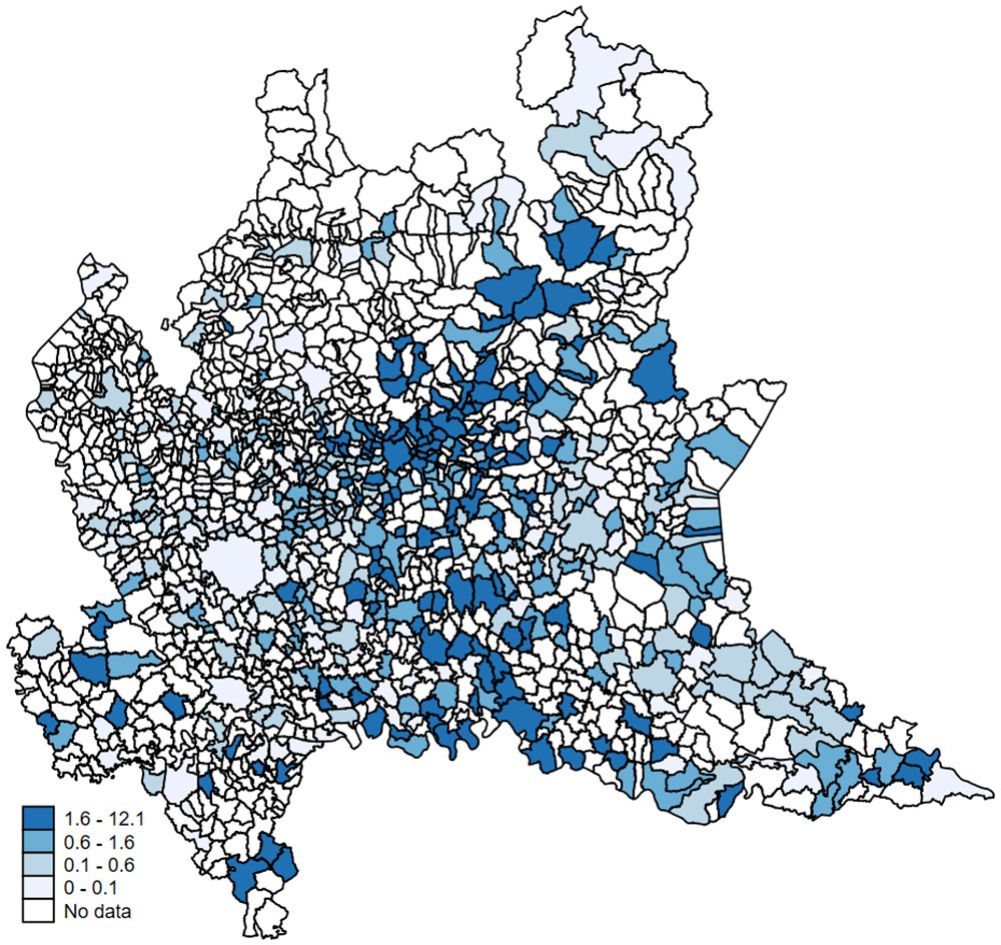
Geographic distribution of unexplained excess mortality (by municipality). *Notes*: This map reports, for each municipality, the geographic distribution of the unexplained excess mortality rate (i.e. the observed excess mortality between March 2020 and March 2019 minus the number of reported COVID-19 deaths as detected by the health authorities) for 1,000 inhabitants. Darker shades of blue refer to municipalities with higher rates of unexplained excess mortality. Authors’ elaboration of publicly available data.

Narrowing down our focus, Fig 2 reports the distribution of mortality rates for Lombardy’s provinces. The difference between the COVID-19 –associated deaths and our measure of excess deaths is glaring and observed across provinces. Fig 3 reports the geographic distribution of unexplained excess mortality rates for each municipality in Lombardy. Darker shades of blue refer to municipalities with higher rates of unexplained excess mortality. A coarse look at Fig 3 suggests the existence of a strong spatial correlation between the most hit municipalities, in line with the hypothesis that COVID-19 spreads through human-to-human close interaction.

**Why under-reporting?**. There are several possible explanations for this form of under-reporting observed in the recorded number of COVID-19–associated deaths. A first may be attributable to the higher fatality ratio in older age groups, in line with [2], a finding that we also point out in Section 3.4. Further, infected patients may die at home without having the ability to be hospitalized and therefore be tested. Presumably, this issue exacerbates for older patients, who are more likely to have a bad pre-existent clinical condition. Connected to this issue, the mere capacity constraint on the number of tests that laboratories can run per day may compromise the correct assessment of the total number of infected patients. This evidence suggests that the actual number of COVID-19 –associated deaths and, therefore, the exact number of infected individuals, is much higher than those reported by public authorities. It is worth noting, however, that the residual excess mortality may also be explained by factors other than COVID-19, such as an excessive stress of the public health system. These explanations are consistent with a recent report published by ISTAT on May 4, 2020 available at https://www.istat.it/it/files//2020/05/Rapporto_Istat_ISS.pdf. For instance, has been registered a reduction of hospitalization for acute myocardial infarction in the northern regions of Italy of about 50% compared to March of the previous year [16].

On the other hand, the likelihood of dying for causes unrelated to COVID-19 (e.g. car or workplace accidents, pollution-related diseases) should be substantially lower during a lock-down period. In fatc, on February 23, 2020, the regional authorities mandated a reduction in public activities (e.g. suspension of sport events and concerts, partial or complete shutdown of all non-necessary commercial activities, etc.), resulting in a substantial decrease in traffic density and air pollution according to the European Space Agency (see http://www.esa.int/ESA_Multimedia/Videos/2020/03/Coronavirus_nitrogen_dioxide_emissions_drop_over_Italy#.Xoh12NOLYiI.link).

#### 3.1.1 A closer look: Bergamo and Brescia

Since the data we are analyzing refer to a selected sample of municipalities which experienced an abnormal increase in mortality rates, we use an alternative source of information to exclude potential sample selection biases and focus only on the two mostly hit provinces. Also in this case, we observe an important increase in the number of deaths. At the aggregate level, we report an increase going from 1,635 deaths in March 2019 to 7,680 deaths in March 2020, equivalent to a 370% increase in the average municipality mortality rate compared to 2019 or previous years. The results are comparable when we look at previous years, as the average number of deaths for the period 2012-2018 was 1,619. Therefore, compared to the other provinces, Bergamo and Brescia experienced a much higher increase in the excess of death. Of these 6,045 excess deaths, only 2,948 deaths have been officially linked to COVID-19, implying that COVID-19–related fatalities account approximately only for half of the observed excess deaths. This proportion is similar to the one we find when using the data from ISTAT at the 28th of March.

### 3.2 A tentative estimate of the actual infections

**Fig 4:**
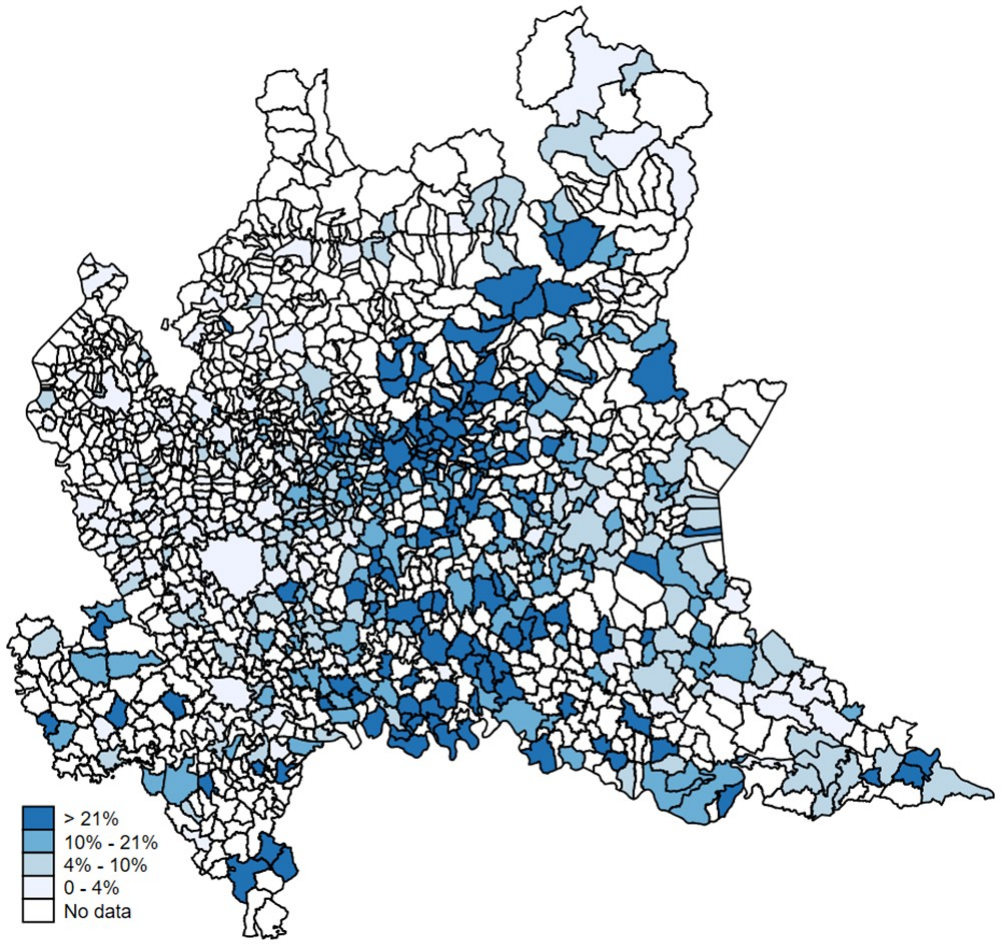
Geographic distribution of hypothetical infection rates (by municipality). *Notes*: For each municipality, this map shows the geographic distribution of the hypothetical share of infected population based on a theoretical mortality rate of 1.5. Darker shades of blue refer to municipalities with higher shares of infected population. Authors’ elaboration of publicly available data.

We report in Table 3 the share of population that is likely to be infected for different fatality ratios (ranging between 1% and 2%) and each Lombardy’s province. In Panel A we report the results for the provinces of Bergamo and Brescia, considering the whole month of March. In the province of Bergamo the share of infected population is estimated to be between 20% and 40%, while in the province di Brescia between 11% and 22%. In Panel B we focus on the data provided by the Italian statistical office. Using these numbers, the real share of infected individuals in Lombardy should range between 6% and 12% of the population. Finally, Fig 4 reports the geographic distribution of the hypothetical share of infected population based on a theoretical mortality rate of 1.5. Darker shades of blue refer to municipalities with higher shares of infected population. Also in this case, darker areas are concentrated around the provinces of Bergamo, Lodi and Cremona and seem to be spatially correlated with each other.

**Table 3:** Share of population likely to be infected, by province.

**Panel A**. *Data at the 31st of March*											
	Hypothetical COVID-19 fatality rate										
*Province*	1%	1.1%	1.2%	1.3%	1.4%	1.5%	1.6%	1.7%	1.8%	1.9%	2%
Bergamo	41.88%	38.07%	34.90%	32.21%	29.91%	27.92%	26.17%	24.63%	23.27%	22.04%	20.94%
Brescia	22.56%	20.51%	18.80%	17.36%	16.12%	15.04%	14.10%	13.27%	12.53%	11.87%	11.28%
**Panel B**. *Data at the 28th of March*											
	Hypothetical COVID-19 fatality rate										
*Province*	1%	1.1%	1.2%	1.3%	1.4%	1.5%	1.6%	1.7%	1.8%	1.9%	2%
Bergamo	40,52%	36,84%	33,77%	31,17%	28,94%	27,01%	25,33%	23,84%	22,51%	21,33%	20,26%
Brescia	18,96%	17,23%	15,80%	14,58%	13,54%	12,64%	11,85%	11,15%	12,07%	9,98%	9,48%
Como	4,69%	4,27%	3,91%	3,61%	3,35%	3,13%	2,93%	2,76%	2,99%	2,47%	2,35%
Cremona	35,75%	32,50%	29,79%	27,50%	25,54%	23,83%	22,34%	21,03%	22,77%	18,82%	17,88%
Lecco	9,63%	8,75%	8,02%	7,41%	6,88%	6,42%	6,02%	5,66%	6,13%	5,07%	4,81%
Lodi	28,91%	26,28%	24,09%	22,24%	20,65%	19,27%	18,07%	17,00%	18,41%	15,21%	14,45%
Mantova	10,62%	9,65%	8,85%	8,17%	7,58%	7,08%	6,64%	6,25%	6,76%	5,59%	5,31%
Milano	4,81%	4,37%	4,01%	3,70%	3,44%	3,21%	3,01%	2,83%	3,06%	2,53%	2,41%
Monza	6,29%	5,72%	5,24%	4,84%	4,49%	4,19%	3,93%	3,70%	4,01%	3,31%	3,15%
Pavia	14,76%	13,42%	12,30%	11,36%	10,55%	9,84%	9,23%	8,68%	9,40%	7,77%	7,38%
Sondrio	7,84%	7,13%	6,53%	6,03%	5,60%	5,23%	4,90%	4,61%	4,99%	4,13%	3,92%
Varese	3,47%	3,16%	2,90%	2,67%	2,48%	2,32%	2,17%	2,04%	2,21%	1,83%	1,74%

*Notes*: This table reports, for each province, the share of population that is likely to be infected for a range of different hypothetical COVID-19 fatality rate. In Panel A we report the estimates using the data collected by newspapers of a sample of 332 municipalities of the province of Bergamo and Brescia covering, respectively, 71% and 95% of the total province population. In Panel B estimates are based on a sample of 548 municipalities provided by the Italian statistical office, covering 67% of the regional population.

### 3.3 An alternative source of data: Obituaries and death notices

**Fig 5:**
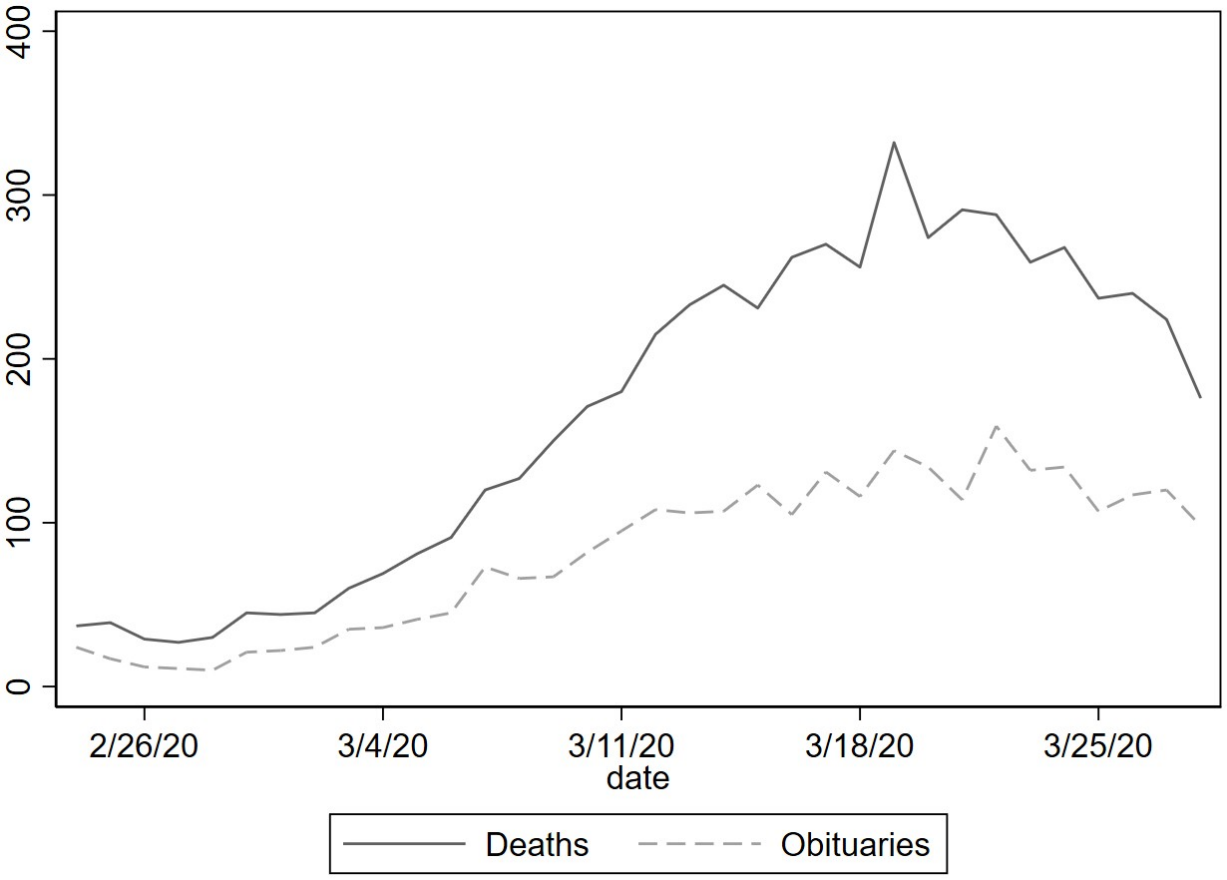
Daily numbers of official deaths and obituaries in the province of Bergamo. *Notes*: Data compares the daily numbers of official deaths published by ISTAT to the daily obituaries and death notices published by L’Eco di Bergamo from February 24, 2020 to April 11, 2020.

Because mortality data may not be immediately available during a pandemic peak, we also use obituaries or death notices published in local newspapers as an alternative source for measuring the number of deaths. Obituaries always contain individual characteristics such as name, surname, age, gender, date of death and place of death (municipality) representing an important and additional source of information to understand the phenomenon better. In March 2019 the number of total deaths registered by official statistics in the entire province was 886 less than one-third of deaths reported this March in newspaper obituaries. Summary statistics show that the average age is 80.9 years, and males represent 59.8% of fatalities. Moreover, looking at the distribution by age group we observe that 0.55% is between 15-49 years, 2.49% between 50-59, 8.42% between 60-69, 27.61% between 70-79, 42.36% between 80-89 and finally 18.54% has more 90 or more years. There has been a significant increase in the daily number of published obituaries after the first week of March being constantly over 100, while during the previous year the daily number was on average around 20. In Fig 5, we plot the daily evolution of obituaries (dashed line) published from February 24, 2020 to April 11, 2020, as well as the daily number of official deaths (solid line) published by ISTAT. Considering the period going from February 24, 2020 to March 28, 2020 the correlation between official deaths and obituaries is 0.98.

Restricting our analysis to the municipalities in the province of Bergamo for which ISTAT released the official data of mortality, we observed that on average obituaries account for 60% of the total registered deaths in the sample. Using this proportion, we can assume that the true number of deaths at the provincial level is roughly twice the number of obituaries published on the newspaper. Specifically, we can estimate that the total number of deaths in March 2020 is 5,138, while the total number of deaths in March 2019 is 1,476, and the excess mortality with respect to March 2019 equals 3,662 (i.e., more than a 400% increase). Using the same reasoning of section 3.1, these additional deaths are probably attributable to COVID-19.

Considering a fatality rate of 1.4%—which is the fatality ratio adapted to the provincial age structure using [5]—the true number of cases should be 260,000 in the province of Bergamo that has 1.1 million inhabitants. This would imply that 24% of the provincial population was infected. This ratio is not too far away from what we find when calculating the excess of mortality by using the data from ISTAT (28%) or the data from the newspapers’ survey (29%). Considering more conservative estimates of the fatality rate of 1.1% the number of cases raises to 330,000, i.e. 30% of the province’s population. Overall, estimates about the spread of the virus using obituaries and death notices are consistent with what reported in Table 3 where different source of data were used.

### 3.4 Suggesting evidence using individual-level data

We show in this section some graphical results using the “crude” measure of CFR.

Despite the kind of biases we are facing using this measure, we can still provide informative elements to assess the relative risks between different demographic groups. Because of possible issues related with measurement errors and sample selection, we decided no to comment the absolute numbers. In principle, all individuals that belong to the sample of detected positive had faced the same potential selection bias. To account for the possible delay between COVID-19 detection and patient’s recovery or death (i.e., data censoring), we report graphical evidence about the variation of individual death risks over time, starting from the day a patient tested positive, and for different demographic characteristics.

**Fig 6:**
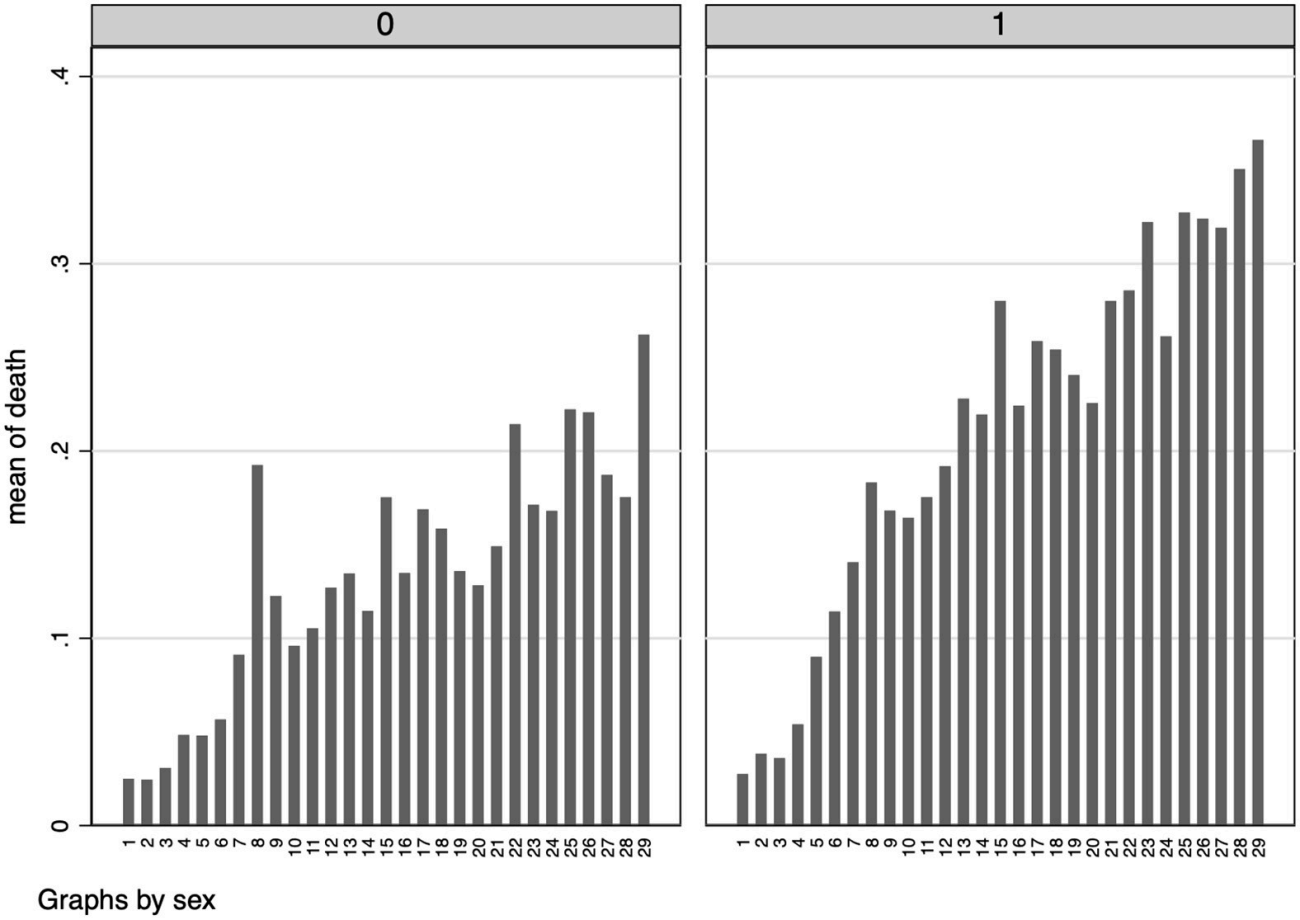
Risk of death (by gender). *Notes*: These graphs report the probability of being dead depending on the number of days since an individual has tested positive to COVID-19, by gender. Females on the left and male on the right.

**Fig 7:**
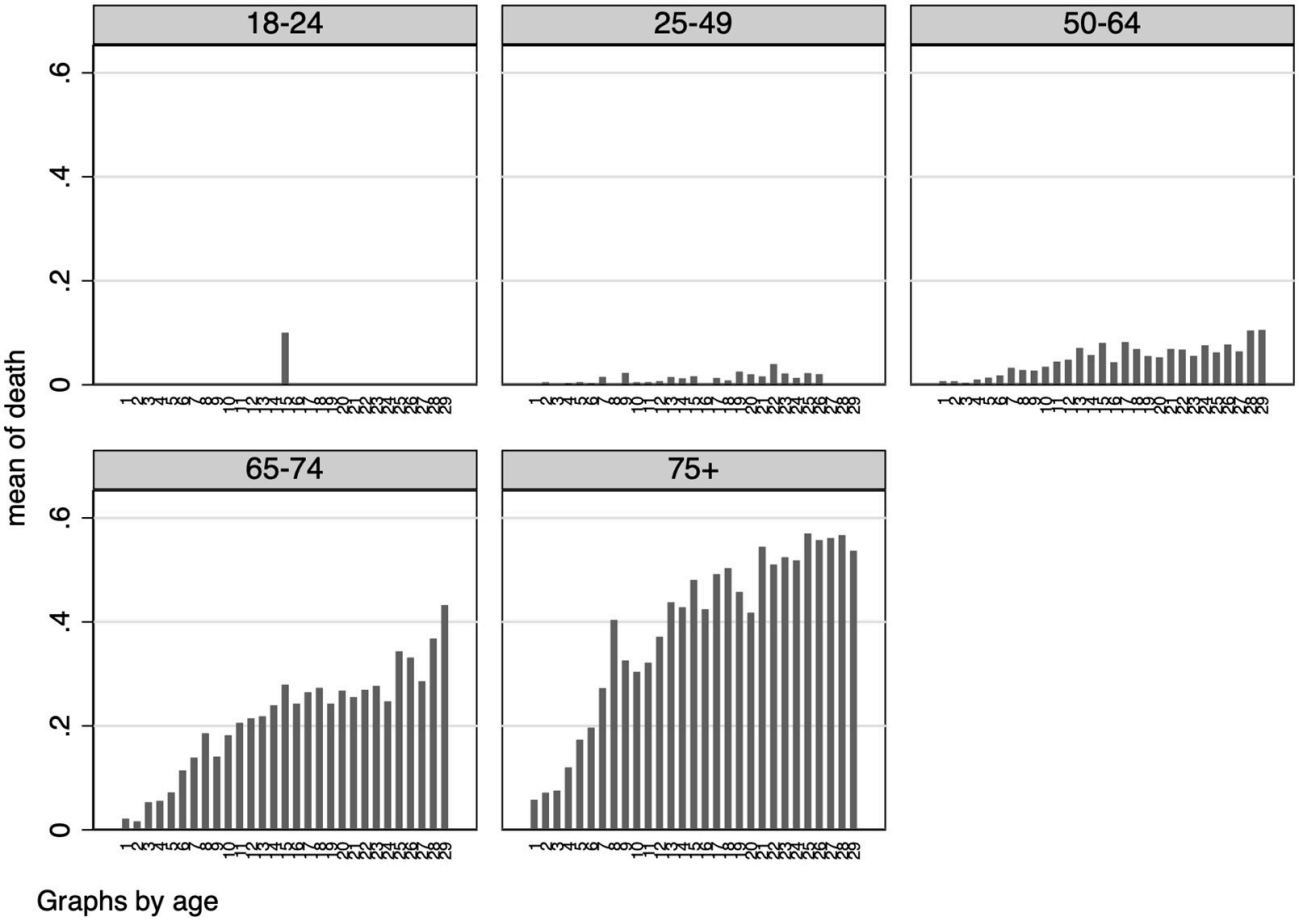
Risk of death (by age group). *Notes*: These graphs report the probability of being dead depending on the number of days since an individual has tested positive to COVID-19, by age group.

In Fig 6 we report such results by gender, while in Fig 7 we focus on different age groups. In line with previous studies—e.g. [2]—we find a higher severity of the disease in older age groups as well as in male individuals. Also, individual death risk increase over time since the day of COVID-19 detection.

## 4 Concluding remarks

Since the spread of the novel COVID-19 emerged in Lombardy in early February 2020, thousands of patients have died, especially in the Province of Bergamo. Understanding the diffusion and assessing the fatality rate of COVID-19 is crucial for implementing effective public and health policies in tackling the disease. Unfortunately, public authorities’ reporting and statistics significantly underestimated the “true” numbers of cases and COVID-19–associated deaths since there exists a vast proportion of infected patients who are not detected. Such underestimation may be the result of the policy adopted by the Lombardy region authorities, mandating that only patients who experience severe clinical symptoms are eventually tested with reliable diagnostic methods (e.g. nasopharyngeal swab) because of the limited capacity of the microbiology laboratories during the pandemic peak. Since February 26, 2020 until now, the Lombardy health authorities prescribe testing only those patients who experience symptoms or who have been in contact with an infected patient [1].

In this contribution, combining official statistics, retrospective and original data we gauge the reliability of official data published during a pandemic peak by combining multiple data sources and providing a tentative estimate of the “true” number of COVID-19 –associated deaths, as well as the total number of persons infected. Our estimates, not free from limitations, suggest that the reported mortality rate attributable to COVID-19 accounts only for one half of the observed excess mortality rate between March 2020 and March 2019. However, part of the residual excess mortality may also be explained by factors other than COVID-19, such as an excessive stress of the public health system.

## Supporting information

S1 Data (ZIP)
